# Whole gene analysis of a genotype G29P[6] human rotavirus strain identified in Central African Republic

**DOI:** 10.1186/s13104-021-05634-4

**Published:** 2021-05-31

**Authors:** Virginie Banga-Mingo, Mathew D. Esona, Naga S. Betrapally, Rashi Gautam, Jose Jaimes, Eric Katz, Diane Waku-Kouomou, Michael D. Bowen, Ionela Gouandjika-Vasilache

**Affiliations:** 1 Laboratoire Des Virus Entériques/Rougeole, Institut Pasteur de Bangui, Ave de L’Indépendance, BP 923, Bangui, Central African Republic; 2 Viral Gastroenteritis Branch, Division of Viral Diseases, NCIRD, CDC, 1600 Clifton Road, NE, Atlanta, GA 30329 USA

**Keywords:** RVA, Whole genome analysis, Central African Republic

## Abstract

**Objective:**

Rotavirus A (RVA) remains the main causative agent of gastroenteritis in young children and the young of many mammalian and avian species. In this study we describe a RVA strain detected from a 6-month-old child from Central African Republic (CAR).

**Results:**

We report the 11 open reading frame sequences of a G29-P[6]-I2-R2-C2-M2-A2-N2-T2-E2-H2 rotavirus strain, RVA/Human-wt/CAR/CAR91/2014/G29P[6]. Nine genes (VP1–VP3, VP6, NSP1–NSP5) shared 90–100% sequence similarities with genogroup 2 rotaviruses. Phylogenetically, backbone genes, except for VP3 and NSP4 genes, were linked with cognate gene sequences of human DS-1-like genogroup 2, hence their genetic origin. The VP3 and NSP4 genes, clustered genetically with both human and animal strains, an indication genetic reassortment human and animal RVA strains has taken place. The VP7 gene shared nucleotide (93–94%) and amino acid (95.5–96.7%) identities with Kenyan and Belgian human G29 strains, as well as to buffalo G29 strain from South Africa, while the VP4 gene most closely resembled P[6]-lineage I strains from Africa and Bangladesh (97%).

## Introduction

Rotavirus A (RVA) is a major cause of diarrhea in infants and young animals worldwide [1]. The RVA genomic classification nomenclature is based on all 11 segments of double-stranded RNA (dsRNA) that encode for six structural proteins (VP1–VP4, VP6, and VP7) and five or six non-structural proteins (NSP1–NSP5/6) [1]. The genotypes of the VP7,VP4,VP6,VP1,VP2,VP3, NSP1, NSP2, NSP3, NSP4, and NSP5 genes are designated G*x*-P[*x*]-I*x*-R*x*-C*x*-M*x*-A*x*-N*x*-T*x*-E*x*-H*x*, where *x* indicates the genotype number [2]. For a majority of human RVA strains, disparity in the backbone genes can be differentiated by three genotype patterns, the Wa-like genogroup 1 (I1-R1-C1-M1-A1-N1-T1-E1-H1), the DS-1-like genogroup 2 (I2-R2-C2-M2-A2-N2-T2-E2-H2), and the AU-1-like genogroup 3 (I3-R3-C3-M3-A3-N3-T3-E3-H3) that are believed to have originated from porcine, bovine, and feline RVAs, respectively [3]. To date, 36 G, 51 P, 26 I, 22 R, 20 C, 20M, 31 A, 22N, 22T, 27 E and 22 H genotypes have been identified in human and non-human hosts and classified based on differences in the nucleotide sequence identities of each encoding gene segment, respectively [3]; http://rega.kuleuven.be/cev/viralmetagenomics/virus-classification. This classification system has accelerated the comparison of RVA genotypes and increased our understanding of the genetic diversity of RVA. Interspecies transmission and genetic reassortment between human and animal RVA have been frequently described, and pigs and cattle are considered the major reservoirs for the genetic and antigenic diversity of human RVA strains.

RVA genotype G29 is rare and has been reported only twice in humans and once in animal previously. The first human strain RVA/Human-wt/BEL/BEF06018/2014/G29P[41] with genetic constellation of G29P[41]-I2-R2-C2-M2-A3-N2-T6-E2-H3 was detected in Belgium in 2013 [4]. The second human strain RVA/Human-wt/KEN/0279/2010/G29P[X] was detected in Kenya in 2010 and only the VP7 gene of this strain has been sequenced [5]. Finally, a single animal strain, RVA/Buffalo-wt/ZAF/4426/2002/G29P[14] with genetic constellation of G29-P[14]-I2-R2-C2-M2-A11-N2-T6-E2-H3 has been detected in a South African buffalo [6]. Here we report the full gene molecular characterization of a novel human G29P[6] RVA strain from the Central African Republic (CAR).

## Main text

### Methods

#### Patient and sample collection

In 2014, a 6-month-old child was hospitalized for acute diarrhea with fever and vomiting at the Complexe Pédiatrique, Bangui (CPB), CAR. The patient had no record of exposure to animals. Diarrheic stool specimen was collected and tested at surveillance site laboratory for RVA antigen by using the Rotaclone EIA kit (Premier Rotaclone™, Meridian Diagnostics, Cincinnati, OH, USA), according to the manufacturer’s instructions. RVA-positive stool specimen was shipped on ice packs to the Centers for Disease Control and Prevention (CDC) for genotyping and sequencing analyses.

#### RVA dsRNA extraction, RT-PCR, sequencing and genotype assignment

RNA was extracted from stool using the QIAamp Viral RNA mini kit according to the manufacturer’s instructions (Qiagen, Valencia, CA, USA). The sequencing templates were prepared using sequence independent whole-genome reverse transcription-PCR (RT-PCR) amplification [7] with slight modifications. The amplified cDNA amplicons were sequenced using the Illumina MiSeq reagent kit v.2, 500 cycles and the standard 250 bp paired-end reads method. Illumina sequence reads were analyzed using CLC Genomics Workbench 11.0 (http://www.clcbio.com/products/clc-genomics-workbench/) incorporating a combination of de novo- and reference-guided assemblies to generate contigs and consensus sequences to obtain the complete open reading frame (ORF) sequences of strain CAR91/2014. Genotypes were determined using RotaC 2.0 (http://rotac.regatools.be/) [8].

#### Phylogenetic and genetic analyses

For each gene, multiple alignments were made by using the MUSCLE algorithm implemented in MEGA6 software [9], http://www.megasoftware.net/). Once aligned, the DNA Model Test program implemented in MEGA version 6 was used to identify the optimal evolutionary models that best fit the sequence datasets. Using Corrected Akaike Information Criterion (AICc), the following models were found to best fit the sequence data for the indicated genes: GTR + G + I (VP1, VP2, VP3, VP4, VP6, VP7, NSP1, NSP2, and NSP3), GTR + G (NSP4), and HKY + G (NSP5). With these models, maximum-likelihood trees were constructed using MEGA 6 with 1000 bootstrap replicates to estimate branch support. Nucleotide and amino acid distance matrices were prepared using the *p*-distance algorithm of MEGA 6 software [9].

### Results

For RVA strain RVA/Human-wt/CAR/CAR91/2014/G29P[6] (henceforth called CAR91/2014), the length of the open reading frames (ORF) for gene segments 1–11 was 3265, 2640, 2508, 2328, 1461, 1194, 933, 954, 978, 494, and 603 bp, respectively and the complete genotype constellation was G29-P[6]-I2-R2-C2-M2-A2-N2-T2-E2-H2. The complete ORFs for all 11 genes of strain CAR91/2014 sequences were deposited in GenBank under accession numbers MT163234 to MT163244 for VP7, VP4, VP6, VP1, VP2, VP3, NSP1, NSP2, NSP3, NSP4, and NSP5, respectively.

#### Phylogenetic analyses

**Fig. 1:**
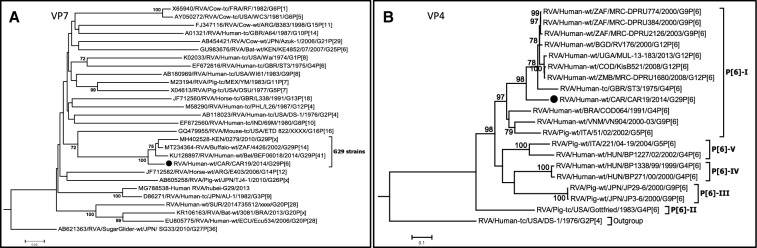
**A**, **B** Phylograms indicating genetic relationships of complete nucleotide sequences of **A** VP7, and **B** VP4 of the study strain RVA/Human-wt/CAR19/2014/G12P[6] from Central African Republic (indicated with a black circle) with representatives of known human and animal rotavirus genotypes. Bootstrap values > 70% are indicated at each branch node. Scale bars indicate the number of nucleotide substitutions per site

**Fig. 2:**
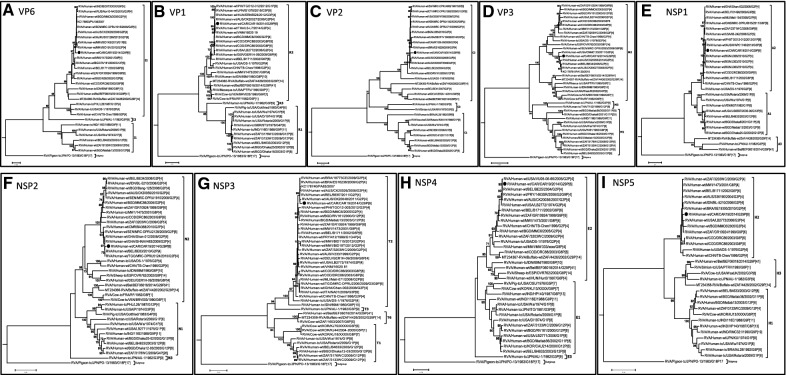
**A**–**I** Phylograms indicating genetic relationships of complete nucleotide sequences of **A** VP6, **B** VP1, **C** VP2, **D** VP3, **E** NSP1, **F** NSP2, **G** NSP3, **H** NSP4 and **I** NSP5 of the study strain RVA/Human-wt/CAR19/2014/G12P[6] from Central African Republic (indicated with a black circle) with representatives of known human and animal rotavirus genotypes. Bootstrap values > 70% are indicated at each branch node. Scale bars indicate the number of nucleotide substitutions per site

Phylogenetic analyses of the eleven gene segments revealed that the VP7 gene of strain CAR91/2014 occupied a basal position in the sub-lineage with two human strains, RVA/Human-wt/BEL/BEF06018/2014/G29P[41] detected in Belgium and RVA/Human-wt/KEN/0279/2010/G29P[X] detected in Kenya, and a single buffalo strain RVA/Buffalo-wt/ZAF/4426/2002/G29P[14] detected in South Africa (Fig. 1A). Nucleotide distance matrices show that the VP7 gene of strain CAR91/2014 shared a moderately high nucleotide (amino acid) identities of 93–94.9% (95.5–96.7%) (data not shown) to cognate sequence of strains RVA/Human-wt/BEL/BEF06018/2014/G29P[41], RVA/Human-wt/KEN/0279/2010/G29P[X] and RVA/Buffalo-wt/ZAF/4426/2002/G29P[14]. The VP4 gene of strain CAR91/2014 clustered in a distinct sub-lineage with African and European human P[6] strains within lineage I of the P[6] genotype (Fig. 1B). Like with the VP7 gene, strain CAR91/2014 occupied a basal position within the sub-lineage. The VP4 gene of strain CAR/91/2014 was most similar (nucleotide 97%, amino acid 98%) to human G9-associated P[6] genes from South Africa and G12-associated P[6] genes from Bangladesh, Zambia, the Democratic Republic of Congo and Uganda (96–97%), all of which belong to previously published P[6]-lineage I [10]. With the exceptions of genes VP3 and NSP4, the remaining genes, VP1, VP2, VP6, NSP1, NSP2, NSP3, and NSP5 cluster closely with almost exclusively human genogroup 2 strains belonging to the R2, C2, I2, A2, N2, T2, and H2 genotypes, respectively (Fig. 2A–I). The only closely related non-human strain sequences were the VP3 and NSP4 genes of ovine, buffalo and cow RVA strains RVA/Sheep-tc/ESP/OVR762/2002/G8P[14], RVA/Buffalo-wt/ZAF/4426/2002/G29P[14] and RVA/Cow-tc/VEN/BRV003/1990/G6P[1]. However, the VP1, VP2, VP3, VP6, NSP1, NSP2, NSP3, NSP4 and NSP5 gene segments of strain CAR91/2014 shared greatest nucleotide (90–100%) and amino acid (93–100%) identities with DS-1-like strains belonging to R2, C2, M2, I2, A2, N2, T2, E2 and H2 genotypes (data not shown).

### Discussion

Here we present the sequence of strain CAR91/2014 which represents the fourth report of the G29 genotype and the first complete sequence for a G29P[6] RVA strain combination detected in humans. A unique feature of this CAR91/2014 strain is that the genes that constitute the genetic backbone belong exclusively to DS-1-like genogroup 2 of human origin, while those of the other human (RVA/Human-wt/BEL/BEF06018/2014/G29P[41]) [4] and animal (RVA/Buffalo-wt/ZAF/4426/2002/G29P[14]) [6] G29 strains with complete sequences displayed the backbone I2-R2-C2-M2-A11-N2-T6-E2-H3,typical of artiodactyl RVA strains. Phylogenetically, the VP7 gene of the strain CAR91/2014 occupied the basal position of the G29 strains, suggesting that the CAR91/2014 might be the ancestral origin of all the G29 genotype that have been detected so far. Analysis of the genetic backbone of the CAR91/2014 strain shows that all the genes, with the exception of the VP3 and NSP4, appears to be human RVA and are phylogenetically linked to human DS-1-like genogroup 2 G9P[6], G12P[6], equine-like G3P[8], reassortant G1P[8] and G3P[4] strains from Australia, Uganda, Brazil, South Africa, Philippines, Thailand, Japan and Hungary. Phylogenetically, the VP3 and NSP4 genes of strain CAR91/2014 clustered together with both human and animal RVA strains, suggesting that the VP3 and NSP4 genes of ovine, buffalo and bovine strains might have originated from a genetic reassortment between human and animal strains. Unfortunately, the complete genome of the Kenyan G29 strain RVA/Human-wt/KEN/0279/2010/G29P[X] was not available for comparison. In a nutshell, the closeness of the genetic backbone of strain CAR91/2014 to those of several DS-1-like strains including reassortant G1P[8] and G3P[8] is an indication that any of these strains might have donated these genes to the CAR91/2014 strain.

Analysis of RVA complete-ORF sequences from all 11 genes provides valuable data to better understand the contemporary diversity among RVA strain and helps to expand our knowledge of the genetic diversity and origin of uncommon RVA genotypes such as CAR91/2014.

## Limitations

This manuscript describe characterization of a single G29P[6] strain and there might be others in circulation in Central African Republic. Also, rotavirus vaccine has not been introduced in Central African Republic, hence no post vaccine data is included in this study.

## Data Availability

The datasets used and/or analyzed during the current study are available from the corresponding author on reasonable request.

## Abbreviations

RVA: Rotavirus group A
CAR: Central African Republic
ORF: Open reading frame
EIA: Enzyme linked Immunosorbent Assay
RT-PCR: Reverse Transcription-Polymerase Chain Reaction
